# Proteasome Inhibition Represses Unfolded Protein Response and Nox4, Sensitizing Vascular Cells to Endoplasmic Reticulum Stress-Induced Death

**DOI:** 10.1371/journal.pone.0014591

**Published:** 2011-01-26

**Authors:** Angélica M. Amanso, Victor Debbas, Francisco R. M. Laurindo

**Affiliations:** Vascular Biology Laboratory, Heart Institute (InCor), University of São Paulo School of Medicine, São Paulo, Brazil; Instituto de Química - Universidade de São Paulo, Brazil

## Abstract

**Background:**

Endoplasmic reticulum (ER) stress has pathophysiological relevance in vascular diseases and merges with proteasome function. Proteasome inhibition induces cell stress and may have therapeutic implications. However, whether proteasome inhibition potentiates ER stress-induced apoptosis and the possible mechanisms involved in this process are unclear.

**Methodology/Principal Findings:**

Here we show that proteasome inhibition with MG132, per se at non-lethal levels, sensitized vascular smooth muscle cells to caspase-3 activation and cell death during ER stress induced by tunicamycin (Tn). This effect was accompanied by suppression of both proadaptive (KDEL chaperones) and proapoptotic (CHOP/GADD153) unfolded protein response markers, although, intriguingly, the splicing of XBP1 was markedly enhanced and sustained. In parallel, proteasome inhibition completely prevented ER stress-induced increase in NADPH oxidase activity, as well as increases in Nox4 isoform and protein disulfide isomerase mRNA expression. Increased Akt phosphorylation due to proteasome inhibition partially offset the proapoptotic effect of Tn or MG132. Although proteasome inhibition enhanced oxidative stress, reactive oxygen species scavenging had no net effect on sensitization to Tn or MG132-induced cell death.

**Conclusion/Relevance:**

These data indicate unfolded protein response-independent pathways whereby proteasome inhibition sensitizes vascular smooth muscle to ER stress-mediated cell death. This may be relevant to understand the therapeutic potential of such compounds in vascular disease associated with increased neointimal hyperplasia.

## Introduction

Endoplasmic reticulum (ER) stress, an important pathophysiological component of diseases such as cancer, diabetes mellitus, neurodegeneration and atherosclerosis, triggers complex specific cell signaling known as the Unfolded Protein Response (UPR) [1]–[3]. The UPR is primarily adaptive and aimed to restore ER homeostasis, but can, if ER stress is intense/sustained or if adaptation fails, lead itself to apoptosis via specific pathways such as those involving transcription factor CHOP/GADD153 [2], [3]. Oxidative stress strongly converges with ER stress in a way that the UPR triggers early reactive oxygen species (ROS) generation, which in turn contributes to sustain proadaptive and/or proapoptotic UPR signaling [4], [5]. Both ER-resident oxidoreductases and mitochondria contribute to such ROS generation [2], [4], [5], but a particular role for Nox4 NADPH oxidase isoform has been reported in vascular smooth muscle cells (VSMC) [5], [6], and in endothelial cells [7]. Mechanisms whereby cell survival is coupled to UPR signaling and ROS generation are yet unclear and appear to be highly variable among distinct cell types [5].

The ubiquitin-proteasome system interfaces with and importantly regulates the UPR. This effect, however, is complex and seemingly ambiguous in a number of aspects. Increased proteasome-mediated degradation of un/misfolded proteins merges with the UPR as an adaptive ER homeostatic mechanism [8], [9], so that proteasome inhibition may potentially lead to ER stress due to lack of removal of damaged proteins [10]. In turn, proteasome inhibitors promote myeloma cell death and disrupt UPR signaling by preventing IRE1α-mediated splicing of the mRNA coding for active transcription factor XBP1, one of the main UPR branches [11]. Also, proteasome inhibition is known to promote oxidative stress [12], but an opposite effect can occur in some cell types [13]. In addition, proteasome function is associated with either cell survival [10], [13], [14] or death [8], [12], [15]–[17], depending on cell type and specific pathophysiological circumstances such as proliferative status. Understanding such questions has become increasingly relevant, given that proteasome inhibition is rapidly emerging as a therapeutic strategy, e.g., against several types of tumors [11], [12], [15].

ER stress and UPR signaling have been shown to mediate several aspects of the pathogenesis and natural history of atherosclerosis and vascular inflammation [1]–[5]. In parallel, the ubiquitin-proteasome system acts as mediator of vascular cell inflammation and survival, by means of NFκB activation and cytokine effects (reviewed in ref. 18). Therefore, vascular effects of proteasome inhibitors have been investigated, with reported evidence suggesting that such compounds have atheroprotective effects and reduce neointima after injury [18]–[22]. However, there is equally substantive information on worsening of atherosclerosis, endothelial function and induction of a rupture-prone plaque phenotype by proteasome inhibition [18], [23], [24]. While such discrepancies appear dependent on model, species, stage of disease and particularly on the degree of proteasome inhibition [25], these controversies indicate that better knowledge of mechanisms underlying effects of the proteasome, as well as proteasome inhibitors, in vascular cells is important in order to provide rational advances. Particularly, mechanisms of proteasome inhibitor effects on vascular cell viability are unclear, specifically those regarding their likely interplay with UPR signaling, oxidative stress and NADPH oxidase.

In this study, we investigated, in VSMC exposed to the classical ER stressor tunicamycin, the role of proteasome inhibition on cell viability, UPR signaling, oxidative stress and NADPH oxidase expression/activity. Our results indicate that proteasome inhibition, per se at non-lethal levels, suppresses ER stress-induced UPR signaling and Nox4 expression, but intriguingly increases XBP1 mRNA splicing. In parallel, proteasome inhibition sensitizes vascular smooth muscle cells to ER stress-induced death, through mechanisms not clearly dependent on ROS.

## Results

### Proteasome inhibition at non-lethal levels potentiate endoplasmic reticulum stress-induced cell death

**Figure 1:**
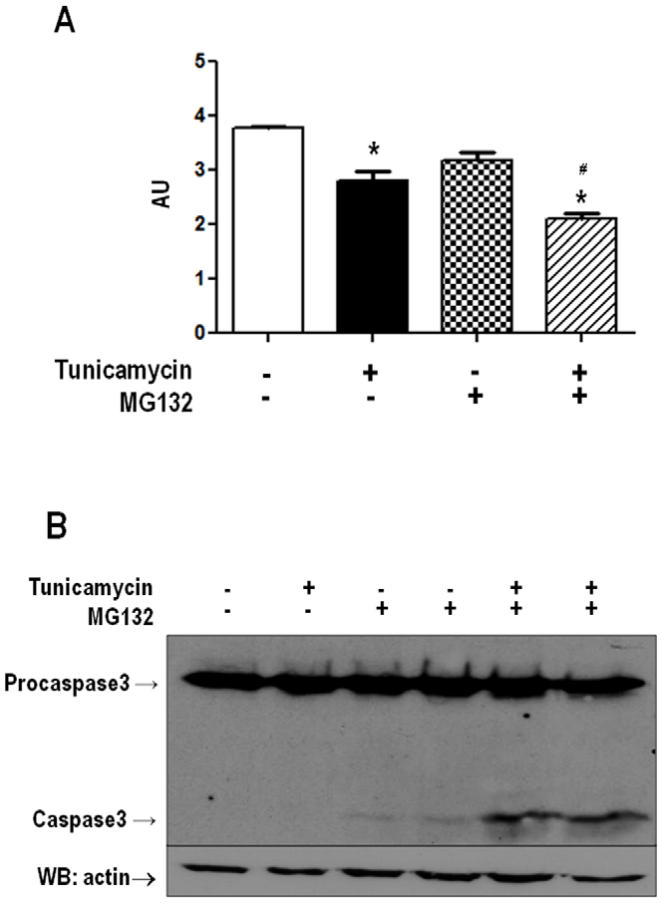
Proteasome inhibition sensitizes VSMC to death due to ER stress. (A) Representative graph of VSMC cell viability by MTT assay. VSMC were incubated with tunicamycin (Tn) (5 µg/mL) or/and MG132 (1 µM) for 16 h, followed by MTT assays. (B) Similar to A, VSMC were incubated with Tn (5 µg/mL) or/and MG132 (1 µM) for 16 hours. Total cell homogenates were submitted to western analysis with anti-caspase-3 antibody. Data are mean ± SD of 3 independent experiments.*P<0.05 vs. Control. #P<0.05 vs.Tn.

**Figure 2:**
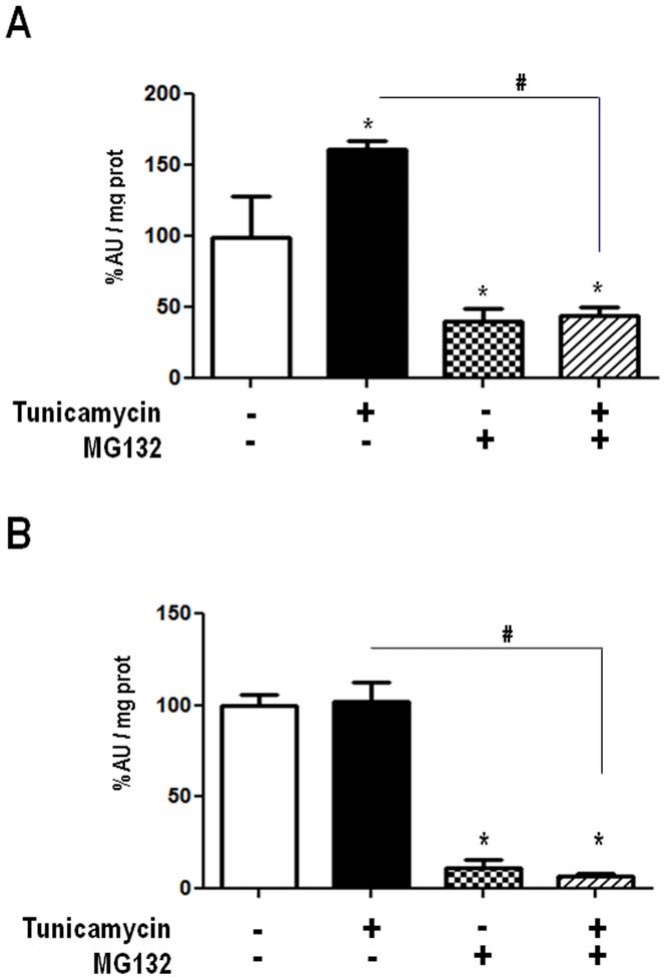
Activity of 20S proteasome in VSMC at control condition or after incubation with AngII (200 nM) or Tn (5 µg/mL), in the absence or presence of MG132 (1 µM) during 4 (A) or 16 (B) hours. Cell lysates were incubated with probe AMC (LLVY-AMC) in the presence of SDS and fluorescence release followed over 15–30 min (excitation 355 nm, emission 460 nm). Data are mean ± SD of 4 or more independent experiments.*P<0.05 vs. Control. #P<0.05 vs. Tn.

Initial experiments were directed to establish concentrations of proteasome inhibitor not associated with cell death within the time frame of our experiments, since cell loss might be accompanied by possible secondary redox and other signaling events. VSMC were incubated in the absence of serum with a range of concentrations of the proteasome inhibitor MG132 (0.1 to 10 µM for 24 h). Cell loss detected by MTT assays started to appear at concentrations equal to or above 3 µM, so that results shown in Fig. 1A reflect data obtained with the 1 µM concentration, chosen for all subsequent experiments. Importantly, such concentration was shown to effectively inhibit proteolytic proteasome activity (Fig. 2). Additional experiments (not shown) showed that the presence of serum provided protection against VSMC death up to 10 µM MG132 concentration. Viability experiments were also performed in presence of the known ER stressor tunicamycin (Tn, 5 µg/mL). Although MG132 at 1 µM concentration was unassociated with significant loss in cell viability, co-incubation of VSMC with Tn plus MG132 induced significant increase in cell loss compared to Tn alone (Fig. 1A). To further assess the effects of MG132 in cell death, western blot experiments were performed against procaspase-3 and caspase-3. Results shown in Fig. 1B indicate a clearly increased cleavage of procaspase-3 with combined incubation of Tn and MG132, in comparison with either compound alone. These data indicate that apoptosis is one mechanism of cell death whereby proteasome inhibition potentiates ER stress-induced lethality.

### Validation of MG132effects regarding proteasome inhibition

**Figure 3:**
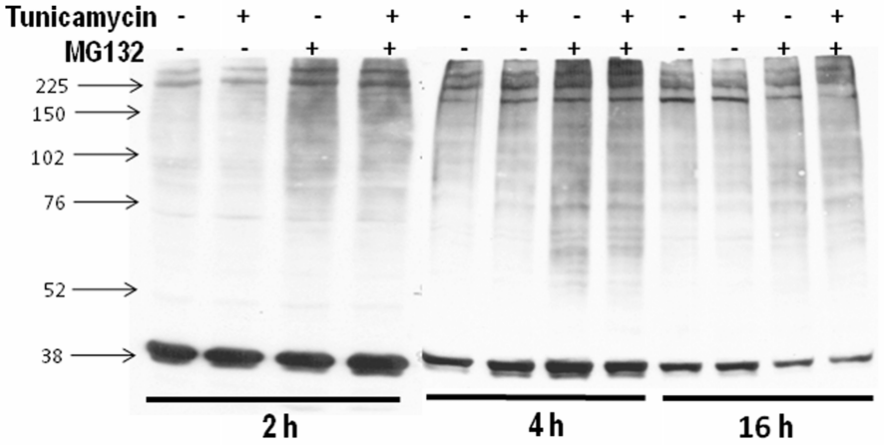
Total polyubiquitinated protein levels after Tn, MG132 or their combination, at 2, 4 or 16 h of incubation with VSMC. Western analysis of VSMC lysates was performed in 8% polyacrilamide gels and probed with anti-ubiquitin antibody. Representative of at least 3 experiments per group.

To assess whether increase in proteasome activity is a normal constituent of the UPR, we assessed 20S proteasome activity (chymotrypsin-like) at several times after starting incubation with Tn. As early as 2 (data not shown) and 4 h of incubation (Fig. 2A), proteasome activity was significantly increased by ∼30%. By 16 h of incubation (Fig. 2B), proteasome activity returned toward baseline levels. Importantly, incubation with MG132 (1 µM) alone or in combination with Tn was capable of significantly inhibiting chymotrypsin-like activity. Since both chymotrypsin-like activity measurement and MG132 action can display some lack of specificity vs. the proteasome, we further assessed the level of total poly-ubiquitinated proteins (Fig. 3) after 2, 4 or 16 h of incubation with Tn, MG132 (1 µM), or their combination. Western analysis showed significant increase in polyubiquitinated proteins, especially at higher molecular weights, at 2 and 4 h (and less evident at 16 h) after MG132, in the absence or not of Tn. The increased chymotrypsin-like activity at 2 and 4 h (Fig. 2A) was not paralleled by detectable decrease in polyubiquitinated proteins, probably because of their already low levels at baseline. Together, these data validate the specificity of MG132 at the 1 µM concentration in our experimental conditions.

### Proteasome inhibition down-regulates UPR signaling

**Figure 4:**
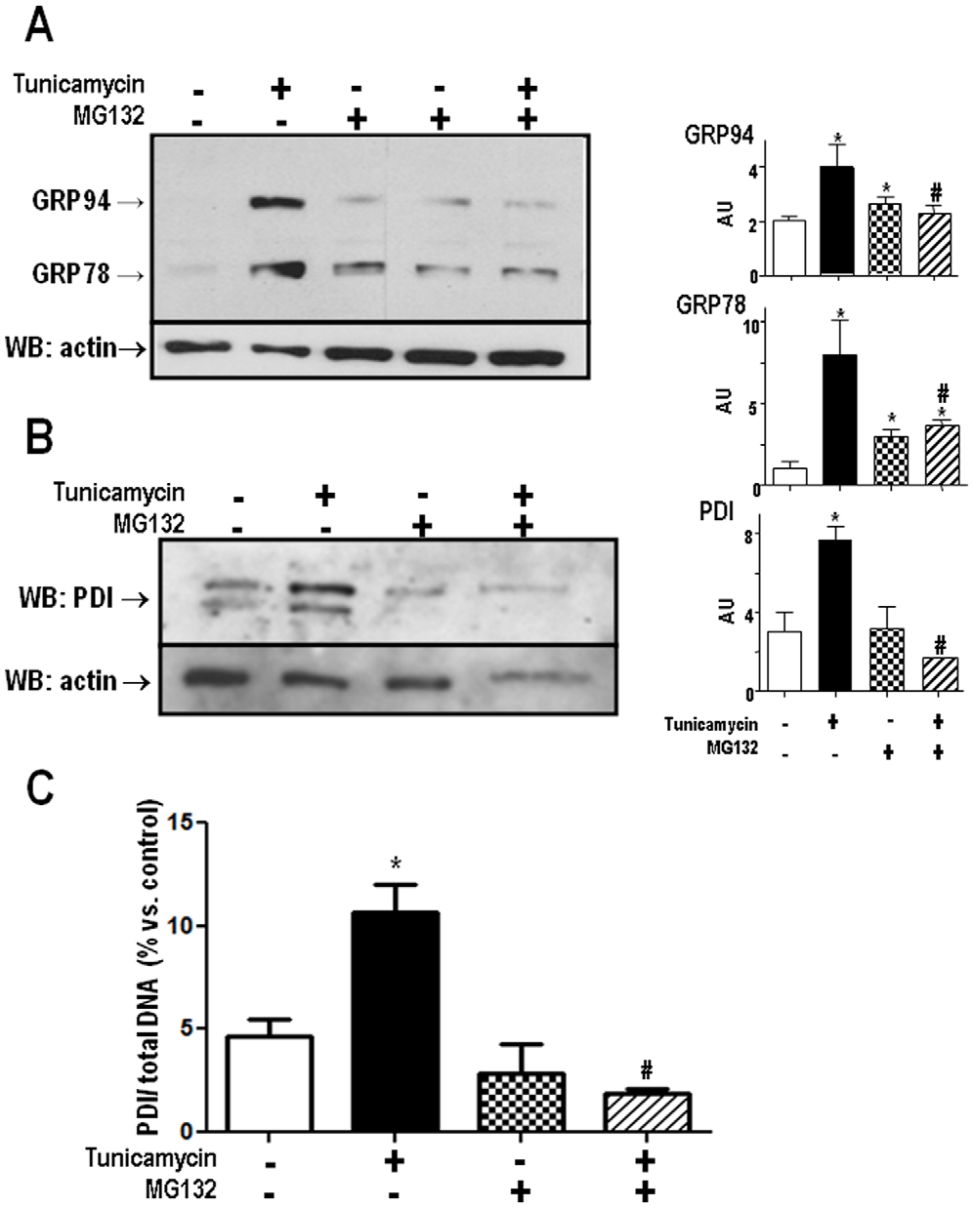
Down-regulation of UPR signaling by MG132. VSMC were incubated with Tn (5 µg/ml), MG132 (1 µM) or their combination for 16 h. Total cell homogenates were submitted to western analysis with anti-KDEL (A) or anti-PDI antibodies (B). Graphs to the right are corresponding densitometric measurements of blots shown in (A) and (B) for at least 3 independent experiments; (C) Analysis of PDI mRNA by real-time PCR. VSMC were incubated with vehicle or Tn (5 µg/mL) in the absence or presence of MG132 (1 µM) for 16 h; n = 3. Data are mean ± SD. *P<0.05 vs. Control. #P<0.05 vs.Tn.

The pattern of early, but not sustained proteasome activity increase during ER stress shown above prompted us to investigate whether the proteasome might be involved in UPR signaling. Incubation of VSMC with the ER stressor Tn (5 µg/mL, 16 h) is known to trigger UPR signaling, including KDEL-containing chaperones such as Grp78 and Grp94, as seen in Fig. 4A. Incubation with MG132 alone promoted mild/moderate increases in the expression of such proteins, while co-incubation of Tn and MG132 significantly prevented their increase in comparison to Tn alone. Under the conditions of our experiments, Tn incubation promoted significant increase in protein expression of the ER redox chaperone protein disulfide isomerase (PDI). While PDI expression was not affected by incubation with MG132 alone (1 µM, 16 h), its co-incubation with Tn completely prevented the increase in PDI protein (Fig. 4B). Real-time PCR analysis of PDI mRNA showed results in line with those depicted for protein expression. VSMC incubation with Tn (5 µg/mL, 16 h) strongly increased PDI mRNA. Again, while MG132 alone was without effect, co-incubation of MG132 with Tn completely prevented the increase in PDI mRNA (Fig. 4C).

**Figure 5:**
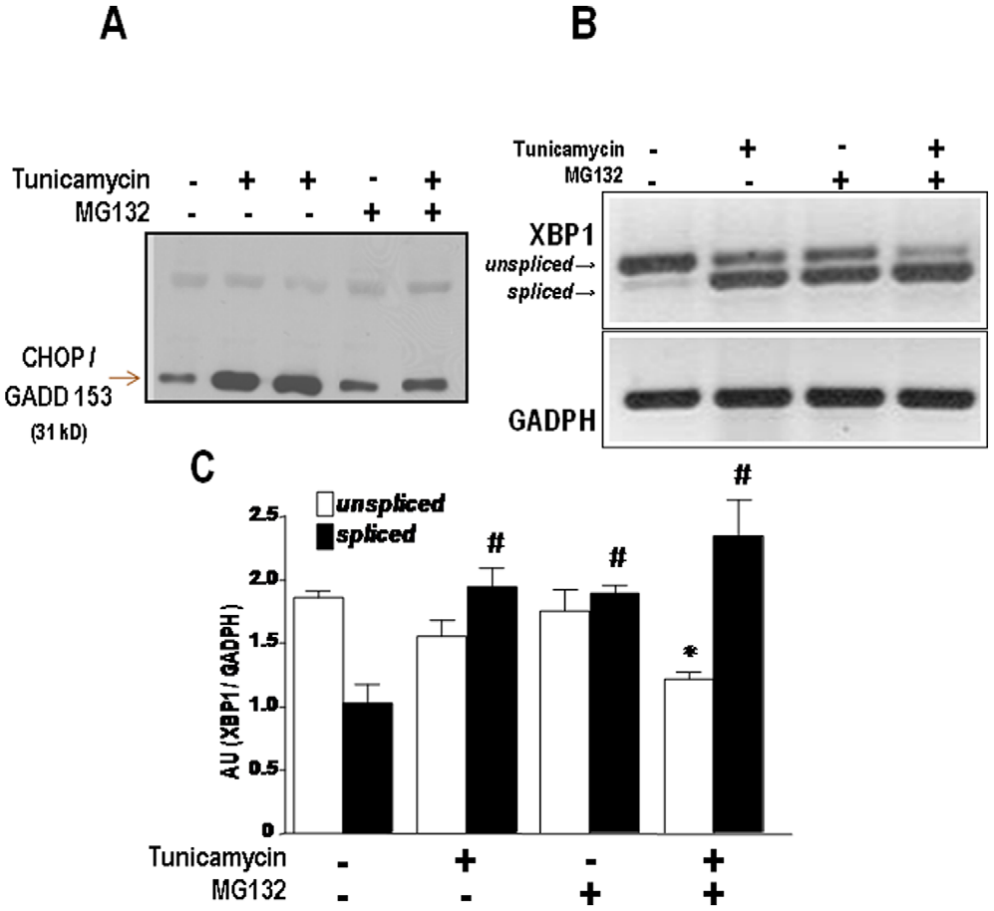
Effects of proteasome inhibition on nuclear CHOP/GADD153 expression and XBP1 mRNA splicing. (A) Representative western analysis of CHOP/GADD153 protein expression in nuclear extracts of VSMC incubated with Tn (5 µg/ml) or/and MG132 (1 µM) for 16 h. (B) Agarose gel depicting the amplified PCR products corresponding to spliced or unspliced forms of XBP1 mRNA, obtained from VSMC submitted to the same conditions as in (A). (C) Graph depicting densitometric analysis of data from (B). Data are mean ± SD of 3 independent experiments. *P<0.05 vs. unspliced control. #P<0.05 vs. spliced control.

Expression of Grp- or PDI-like chaperones represents a major branch of proadaptive signaling during the UPR [2], [3]. To assess whether proteasome inhibition also affects proapoptotic UPR signaling, we assessed the expression of CHOP/GADD transcription factor in the nuclear fraction of VSMC incubated with Tn, MG132 or their combination. Incubation with Tn (5 µg/mL, 16 h) showed the expected strong increase in CHOP expression, consistent with the increase in cell loss verified in Fig. 1A experiments. MG132 incubation (1 µM, 16 h) was without effect, but co-incubation of MG132 (1 µM) and Tn significantly inhibited the nuclear expression of CHOP/GADD 153 (Fig. 5A), indicating that proteasome inhibition suppresses not only proadaptive but also proapoptotic signaling and that potentiation of Tn-induced cell death by proteasome inhibition does not appear to occur through the canonical ER stress-dependent pathway.

### Proteasome inhibition enhances XBP1 splicing

The transcription factor XBP1 has an unusual mode of regulation in which its mRNA is spliced in the cytosolic face of the ER by the endonuclease action of IRE1α, with removal of an intronic sequence, generating the mRNA for the active spliced transcription factor, while the unspliced form acts as a dominant-negative UPR inhibitor [2], [29]. The spliced form of XBP1 mRNA generates a transcription factor that codes for proteins that account for a number of functions that converge to cell survival and ER homeostasis, DNA repair, cell differentiation [30] and protection against oxidative stress [31]. In myeloma cells, MG132 strongly inhibits XBP1 splicing and disrupts UPR signaling [11]. Analysis of XBP1 mRNA splicing in our VSMC through RT-PCR showed the expected robust increase in the spliced form with Tn (5 µg/mL, 16 h). Surprisingly, however, MG132 alone and particularly in combination with Tn also promoted a strong increase in XBP1 splicing (Fig. 5B and C). Strong activation of such UPR survival pathway further suggests that MG132-induced increase in Tn-triggered apoptosis occurred through pathways unrelated to canonical UPR proapoptotic signaling.

### Phosphorylation of Akt and p38 MAPK after proteasome inhibition

**Figure 6:**
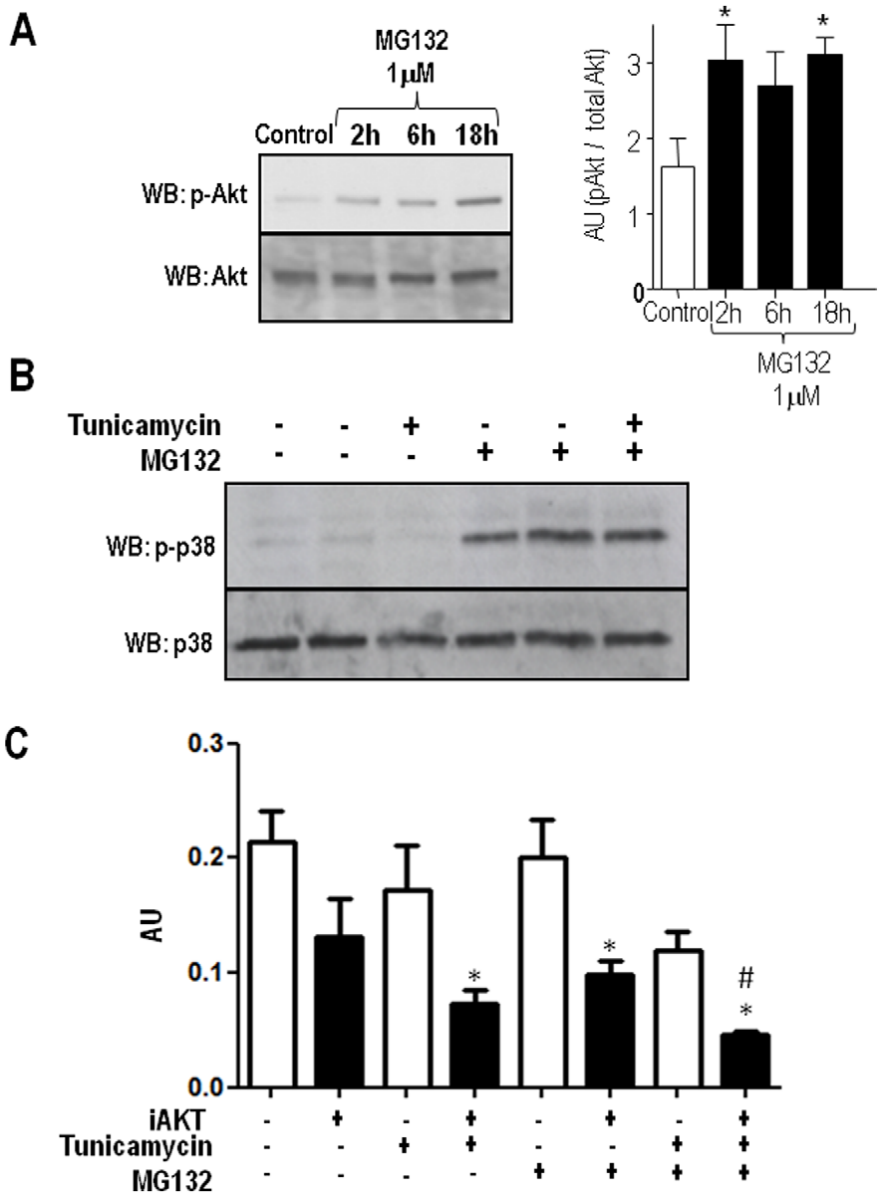
Akt and p38 phosphorylation by MG132 in VSMC. (A) Representative immunoblotting depicting phosphorylated and total Akt expression at baseline or after incubation with MG132 (1 µM) for 2, 6 or 16 h. Graph to the right depicts quantitative densitometric analysis of Akt expression from 3 blots similar to (A). (B) Representative immunoblotting depicting phosphorylated and total p38MAPK expression at baseline or after incubation with angiotensin II, tunicamycin, MG132 (1 µM, 16 h) or their combination; representative of n = 3. (C) Graph summarizing the effect of Akt inhibition on enhancement of VSMC death. VSMC were incubated with Tn (5 µg/mL) or/and MG132 (1 µM) for 16 h in the absence or presence of Akt inhibitor (A6730 Sigma-Aldrich), followed by MTT assays. Data are mean ± SD of 3 independent experiments.*P<0.05 vs. Control. #P<0.05 vs.Tn.

To further investigate the consequences of proteasome inhibition in downstream signaling pathways involved with survival and stress, we assessed Akt/protein kinase B and p38 phosphorylation. VSMC incubated with MG132 (1 µM) for time periods varying from 2 - 18 h showed sustained increases in Akt phosphorylation (Fig. 6A). For p38, MG132 incubation times of 0.5, 4 and 16 h also showed persistent increase in phosphorylation, not detectably time-dependent (not shown). In additional experiments (Fig. 6B), VSMC were incubated for 16 h with AngII (200 nM, used as a positive control) or Tn (5 µg/mL), showing no increase in p38 phosphorylation. Contrarily, incubation with MG132 (1 µM) alone showed that p38 phosphorylation was significantly increased, while MG132 co-incubation with AngII or Tn induced no further detectable changes.

We next investigated the importance of Akt in cell death induced by Tn, MG132 or their combination. For these studies MTT assay was performed in VSMC incubated with Tn (5 µg/mL) and/or MG132 (1 µM) for 16 h in the presence or absence of an Akt inhibitor (A6730 Sigma-Aldrich). Results showed that Akt inhibition increased cell death vs. control already at baseline conditions, but was particularly lethal after Tn, MG132, or their combination. Also, such increase in cell death was greater with the co-incubation of Tn and MG132 in comparison with Tn alone. This indicates that Akt significantly contributes to cell survival during either ER stress or proteasome inhibition in an additive way.

### Proteasome inhibitor induces ROS production and disrupts NADPH oxidase activity

**Figure 7:**
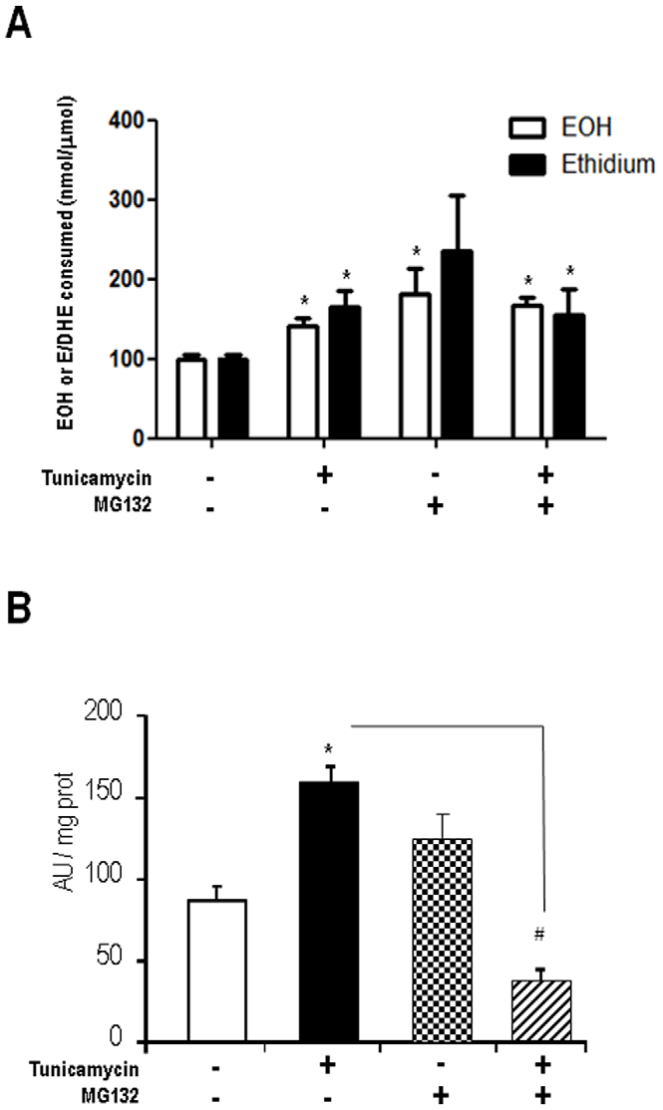
Proteasome inhibition induces ROS production and disrupts ER stress-induced NADPH oxidase up-regulation. (A) ROS production in VSMC incubated for 4 h with Tn (5 µg/mL) or/and MG132 (1 µM), assessed through HPLC analysis of DHE oxidation products (50 µM, 30 min incubation), as described in Methods. Results depict levels of 2-hydroxyethidium (EOH) or ethidium (E) products. (B) NADPH oxidase activity measured in membrane-enriched homogenates from VSMC incubated for 4 h with Tn or MG132. Activity was measured with DHE technique, analogous to (A), as described in Methods. Data are mean ± SD of 3 independent experiments.*P<0.05 vs. Control. #P<0.05 vs.Tn.

Since ROS production and NADPH oxidase activity are integral components of the UPR, we investigated effects of proteasome inhibition on these variables. Experiments to assess ROS production were performed at 4-h incubation periods, in order to detect early changes that would be likely independent of secondary signaling events, as well as apoptosis itself [5]. Generation of 2-hydroxyethidium and ethidium products of DHE was assessed after 4-h incubation with Tn (5 µg/mL), MG132 (1 µM) or their combination (Fig. 7A). Either Tn or MG132 induced increase in ROS signals, while their combination was not additive. Reports from other [6], [7] and our [5] laboratories showed previously that ER stress induces Nox4 mRNA expression in VSMC and that such induction is able to modulate either proadaptive or proapoptotic signaling [5]–[7]. We first assessed the effects of proteasome inhibitors in membrane fraction NADPH oxidase activity. VSMC were incubated for 4 h with Tn, MG132 or their combination and NADPH-driven ROS generation was assessed in membrane fraction with DHE assay (Fig. 7B). Incubation with MG132 alone produced non-significant changes. However, MG132 co-incubation with Tn remarkably switched the response to Tn from 83% increase to 58% decrease in NADPH oxidase activity (Fig. 7B). These results indicate that proteasome inhibition strongly disrupts ER stress-induced NADPH oxidase up-regulation.

### Proteasome inhibition abrogates Nox4 mRNA expression after ER stress

**Figure 8:**
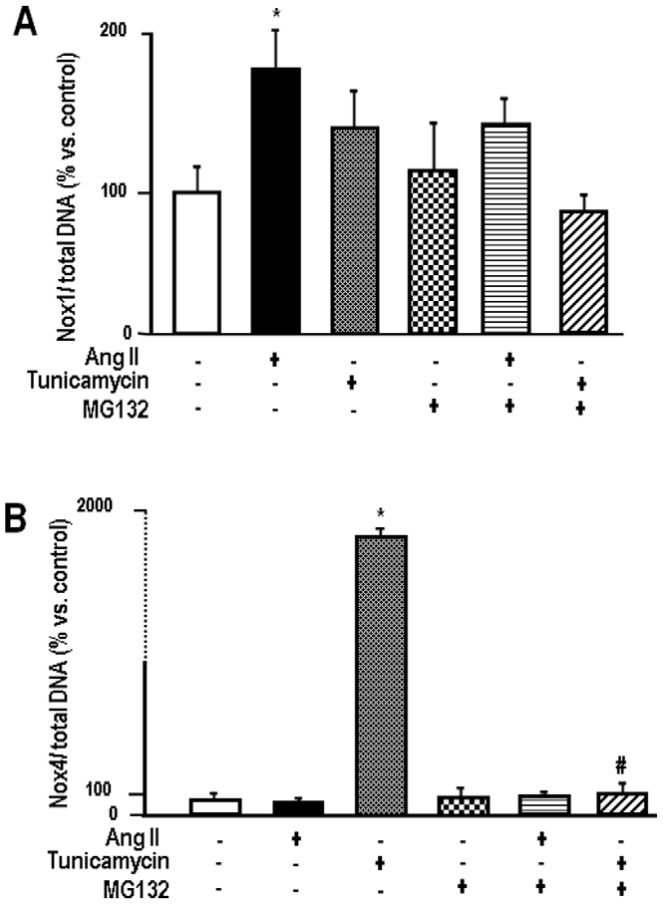
Proteasome inhibition strongly inhibits ER stress-induced Nox4 expression. (A) Real-time PCR analysis of Nox1 mRNA levels. VSMC were incubated with AngII (200 nM) or Tn (5 µg/mL) in the absence or presence of MG132 (1 µM) for 16 h; (B) Similar to (A), with analysis of Nox4 mRNA levels. In both (A) and (B), data are expressed as the ratio of Nox expression/total DNA expression in the same sample. Data are mean ± SD of 5 independent experiments.*P<0.05 vs. Control. #P<0.05 vs. Tn.

NADPH oxidase activation in non-phagocytic cells is characterized by increases in mRNA or protein expression of relevant catalytic or regulatory subunits [32]. Thus, we quantitatively assessed the effects of ER stress and proteasome inhibitor MG132 in mRNA levels of Nox4 and compared them to Nox1, another isoform consistently expressed in VSMC [5], [28], [32] (Fig. 8). Since AngII is a canonical stimulus for Nox1 and does not by itself increase UPR markers in the conditions of our experiment [5], results were also analyzed after VSMC incubation with this peptide. Data are reported for 16-h incubations, since detectable changes in expression tend to occur at later stages, particularly Nox4 after Tn [5]. Additional experiments performed at earlier stages showed increased variability (not shown). MG132 (1 µM) alone induced no evident changes in Nox4, as well as Nox1 mRNA levels. Incubations with AngII (200 nM) or Tn (5 µg/mL) induced preferentially the expected increases in Nox1 or Nox4 mRNA levels, respectively. However, their co-incubation with MG132 significantly altered this expression pattern. Nox1 average increase with AngII was attenuated from 87 to 29% while, remarkably, Nox4 mRNA increase with Tn was essentially abolished after co-incubation with MG132 (Fig. 8B). We also assessed mRNA expression of p22phox in the same conditions as above, but detected no significant changes (data not shown).

### ROS scavenging does not preserve cell viability in ER stressed-VSMC after proteasome inhibition

**Figure 9:**
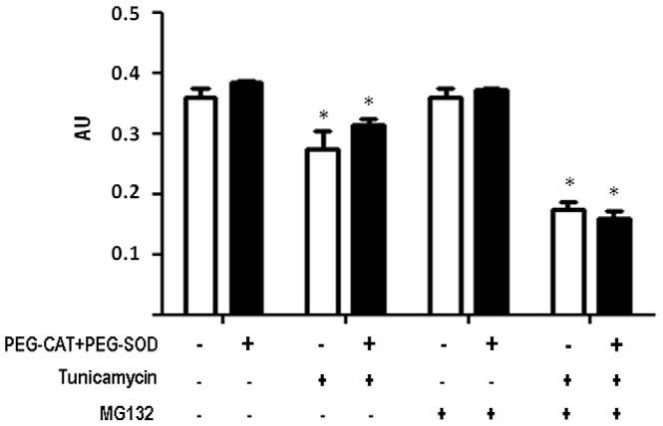
Effects of ROS scavenging in VSMC viability after ER stress or/and proteasome inhibition. VSMC were incubated with Tn (5 µg/mL) or/and MG132 (1 µM) for 16 h in the absence or presence of PEG-Cat (200 U/ml) plus PEG-SOD (25 U/ml). Data are mean ± SD of 3 independent experiments.*P<0.05 vs. Control. #P<0.05 vs.Tn.

To investigate whether ROS played a direct effect on cell viability during proteasome inhibition in ER-stressed VSMC, we co-incubated VSMC with Peg-CAT plus Peg-SOD in the same conditions and then performed MTT assays (Fig. 9). These experiments showed no significant changes in cell viability in the presence of such ROS scavengers.

## Discussion

Our data showed that proteasome inhibition, per se at non-lethal levels, sensitizes VSMC to cell death during ER stress induced by tunicamycin (Tn). This effect was accompanied by suppression of both proadaptive and proapoptotic markers of classical UPR signaling, although, intriguingly, the splicing of XBP1 was markedly enhanced and sustained. In parallel, proteasome inhibition induced complete disruption of NADPH oxidase activity and regulation and of Nox4 mRNA triggered by ER stress. Although oxidative stress was increased, ROS scavenging with PEG−SOD + PEG−CAT had no net effect on VSMC death in our model. Despite the increase in ER stress-induced cell death, proteasome inhibition also promoted survival/stress signaling such as phosphorylation of p38MAPK and Akt, the latter shown to partially offset VSMC death. Together, these data indicate mechanisms of the stress response and redox signaling involved in VSMC cell death induced by proteasome inhibitors, which may be of relevance to understand the therapeutic potential of such compounds in vascular diseases.

The mechanisms whereby proteasome inhibition sensitizes ER-stressed VSMC to apoptosis may be multiple, but a likely major factor was the suppression of homeostatic UPR signaling such as failure to upregulate classical chaperone markers including Grp78, Grp94, as well as PDI. Disruption of UPR signaling is known to sensitize cells to death in response to ER stressors [1], [2], [8], [9], [11]. Such conversion from adaptive to apoptotic signaling, however, likely occurs through distinct mechanisms than those associated with sustained/intense ER stress. While the latter involves canonical UPR pathways such as CHOP/GADD153, the former likely involves UPR-independent pathways, as shown in our study by the lack of activation of such transcription factor. In fact, proteasome inhibitors induce a complex cell stress response, including in some instances UPR signaling itself, which reflects disruption of misfolded protein degradation [8], [18]. In our VSMC, however, this response was quite modest, possibly due to the moderate concentrations of MG132. Proteasome inhibition may also promote reversible arrest in protein synthesis [17], possibly reminiscent of an integrated cell stress response [2]. Another effect of proteasome inhibition may be autophagy [37]. This process, however, was reportedly triggered via UPR-dependent pathways [36], which were suppressed in our VSMC.

The UPR-independent pathways that culminate in executioner caspase-3 activation and apoptosis in our VSMC were likely complex, as in most other studies dealing with proteasome inhibitor-mediated apoptosis. Evidence suggests that these triggering pathways are upstream of mitochondrial dysfunction and caspases, and seem to converge to persistent activation of proapoptotic bcl-2 family proteins[18], [33], [34], inhibition of NF-κB activation [18], or inhibition of FoxM1 transcription factor [35]. In addition, evidence for a negative reciprocal regulatory interaction between the 26S-proteasome and executioner caspases was recently described, suggesting that proteasome inhibition may directly unleash activity of executioner caspases [36]. It is precisely this variety of proapoptotic mechanisms that makes proteasome inhibition a valuable strategy for eluding homeostatic/adaptive pathways responsible for tumor resistance [33] and possibly neointima formation [18], [19].

A frequent [18], [38]–[40] though not universal [13], [14] effect of proteasome inhibition is oxidative stress, associated with ROS generation and in some cases with disrupted mitochondrial membrane potential [15], [40]. Although ROS production was increased in our VSMC, incubation with ROS scavengers SOD and catalase did not significantly prevent sensitization to ER-induced apoptosis by proteasome inhibition, suggesting that the overall mechanism of cell death is not directly related to ROS themselves, or that ROS are consequence rather than cause of the cell death process. Since SOD and catalase were not targeted to specific organelles, we cannot exclude that an early ROS-dependent event in a specific subcellular compartment triggered subsequent ROS-independent apoptotic events that were executed in the cytosol. In addition, it is not inappropriate to propose that these findings can also reflect complex effects of ROS in which both proapoptotic and prosurvival signals are redox-transduced and the overall balance remains neutral. Indeed, Nox4-dependent ROS in the ER were shown to trigger autophagy signaling during ER stress, while Nox4 silencing switched the response to apoptosis [7].

The induction of survival signals such as XBP1 and particularly Akt phosphorylation, concomitant to cell death sensitization by proteasome inhibition is in line with the complex cell stress response triggered by such compounds and significantly limited VSMC loss. The role of Akt in cell survival during ER stress has been described previously [41]–[43] in a way that this kinase may be a key element in the transition from proadaptive to proapoptic signaling. Since in our cells the UPR was largely suppressed by proteasome inhibition, the additive prosurvival effect of Akt in face of proteasome inhibition (Fig. 6C) is likely to involve a distinct, UPR-independent, pathway. It is important to consider that in proliferating cells, proteasome inhibition tends to induce apoptosis, whereas in differentiated cells such as neurons these compounds may reduce apoptosis [39]. This may be relevant to VSMC, which show a high degree of phenotypic modulation during vascular response to injury.

The mechanisms whereby proteasome inhibition suppressed the UPR in our VSMC are unknown. In striking contrast with the other UPR markers assessed in our cells, as well as with findings reported for myeloma cells [11], XBP1 splicing was increased in VSMC after proteasome inhibition, particularly in concomitance with ER stress. XBP1 splicing is an important adaptive component of the UPR, inducing genes coding for chaperones, ER homeostatic proteins, DNA repair and particularly for antioxidant enzymes [30], [31]. In cardiomyocytes, proteasome inhibition promotes the induction of CHOP and ATF6 transcription factors, but not XBP1 or KDEL chaperones [14]. Therefore, it is possible that the response of XBP1, as well as that of UPR signaling in general, to proteasome inhibition is particularly cell-specific. Importantly, the activation of XBP1 in a proapoptotic scenario such as our model is by no means inconsistent with known pathways of UPR regulation. First, although spliced XBP1 has many protective targets, it can also induce the Hsp40 family member p58^IPK^, which may precipitate cell death by releasing PERK-dependent inhibition of protein translation [44]. Second, in a model of genetic deficiency of the anti-apoptotic protein BI-1 (Bax inhibitor-1), which regulates the UPR by inhibitory association with IRE1α, there was marked and persistent XBP1 splicing despite increased sensitivity to ER stress-mediated apoptosis [45], [46]. This highlights the increasing focus on IRE1α as a main regulator of ER stress-related apoptosis [46]. Another proapoptotic regulatory signal converging to this pathway is protein tyrosine phosphatase 1B (PTP-1B), which regulates the UPR via IRE1α activation [47]. Since Nox4, known to be induced by UPR [5]–[7], strongly inhibits PTP-1B [48], [49], one can speculate a model in which suppression of Nox4 by proteasome inhibition allows PTP1-B to potentiate IRE1α and consequently XBP1 activation, within a proapoptotic scenario.

The potential mechanisms of Nox4 repression after proteasome inhibition can be multiple and our data may help understand the intriguing and so far poorly understood regulation of this NADPH oxidase isoform. One possible explanation for this effect is the known transcriptional role of the proteasome, which has been increasingly recognized [50], [51]. In addition to transcription, the mild UPR triggered by proteasome inhibition in VSMC may induce translation repression [17]. Finally, the proteasome may potentially regulate levels of relevant transcription factor proteins [52]. Importantly, the regulation of Nox4 activity appears to strongly correlate with its mRNA levels [53]. Therefore, the effects of proteasome inhibition during the UPR may directly impact on Nox4 activity and ROS production at the oxidase milieu, as in fact reflected in the marked prevention, by MG132, of NADPH oxidase activity upregulation due to Tn. In parallel with Nox4, our results show that proteasome inhibition also impairs mRNA and protein expression of PDI. This is relevant because we showed previously that PDI associates, among other NADPH oxidase subunits, with Nox4 [26], [54]. Down-regulation of PDI prevents ROS generation during the UPR (Santos CX et al, unpublished observations from our laboratory). Very little is known about regulatory mechanisms of PDI transcription in eukaryotes, in part because abundantly expressed PDI seems regulated at the level of protein expression, translocation and perhaps post-translational modifications [54].

In summary, our results indicate that proteasome inhibition impairs VSMC viability during ER stress, while largely suppressing proadaptive and proapoptotic UPR signals, although not XBP1 splicing. The mechanism of such death sensitization likely involves a complex cell stress response induced by proteasome inhibitors, as suggested by the concomitant induction of survival signals such as Akt phosphorylation. In addition, Nox4 as well as PDI induction by ER stress were importantly inhibited by proteasome inhibition. Overall, VSMC death sensitization due to proteasome inhibition did not seem directly dependent on ROS, despite increases in ROS production. ER stress plays a significant role in the pathogenesis of atherosclerotic vascular disease, as well as its complications and risk factors such as hyperlipidemia and insulin resistance [1]. Moreover, such a role is paralleled by interaction between the proteasome and inflammation via NFκB, cytokines and survival pathways [18]. Our results may help understand the involvement of the proteasome in the pathogenesis of vascular disease, as well as clarifying the potential therapeutic role of proteasome inhibitors in atherosclerosis.

## Materials and Methods

### Materials

Antibodies were obtained as follows: PDI and KDEL from Assay Designs/Stressgen (Ann Arbor, MI); CHOP/GADD153 from Thermo Scientific (Golden, CO); Akt, phospho-Akt, p38 and phospho-p38 from Cell Signaling Technology (Danvers, MA); ubiquitin from Zymed/Invitrogen (San Francisco, CA); β-actin from Sigma-Aldrich (St. Louis, MO). Secondary antibodies conjugated with horseradish peroxidase, 20S proteasome activity kit and protease inhibitors were from EMB Biosciences (San Diego, CA, USA). Dihydroethidium (DHE) was from Invitrogen (Carlsbad, CA). All other reagents were from Sigma-Aldrich (St. Louis, MO).

### Cell culture and ER stress induction

Rabbit aortic smooth muscle cells (VSMC) were obtained from a previously established selection-immortalized line, as reported previously [26] and maintained in growth medium (F12+10% fetal bovine serum + streptomycin 100 µM + penicillin 100 U/mL). In specific experiments, VSMC were incubated with angiotensin II (AngII) at a final 200 nM concentration in the absence of FBS. For induction of ER stress, VSMC were incubated for the indicated periods of time with Tunicamycin (Tn) at 5 µg/mL concentration, established by previous experiments to uniformly promote expression of UPR signaling markers in our VSMC [5]. Tn is a classical inducer of ER stress by virtue of its inhibitory effect on N-glycosylation of nascent proteins destined to membranes or extracellular secretion [2]. For proteasome inhibition, VSMC were incubated with MG132 at 1 µM concentration for the indicated periods of time. This concentration was established after preliminary experiments (see Results), in which a wide range of concentrations was tested, and is in line with concentrations used in other studies from the literature [10], [14].

### MTT assays for cell viability

VSMC (2×10^4^ cells) were seeded and cultured for 24 h in a 96-well plate, followed by further incubation for 24 h in presence or absence of Tn or/and proteasome inhibitor MG132 at the indicated concentrations, while during the last 4 h MTT (3-(4,5-dimethylthiazol-2-yl)-2,5-diphenyltetrazolium bromide; 600 µM) was added. VSMC were then washed with PBS (pH 7.4), followed by DMSO, which solubilizes formazan crystals. Absorbance was measured at 570 nm.

### Measurement of ROS levels in VSMC by HPLC analysis of DHE oxidation products

Cells grown in 6-well dishes (well area of 9.6 cm^2^), at 90% confluence, were incubated or not with Tn (5 µg/mL) or MG132 (1 µM) for 16 h. ROS generation was assessed through HPLC analysis of DHE oxidation products, as described in detail previously [27]. Briefly, cells kept in serum were washed twice with PBS and incubated in the dark with PBS/DTPA (0.5 ml) and DHE at final 50 µM concentration for 30 min in the absence of serum. Cells were then washed 2x in cold PBS, harvested in acetonitrile (0.5 ml/well), sonicated (10 s, 1 cycle at 8 W), and centrifuged (12,000 *g* for 10 min at 4°C). Supernatants were dried under vacuum (Speed Vac Plus model SC-110A, Thermo Savant) and pellets maintained at −20°C in the dark until analysis. Samples were then resuspended in 120 µl PBS/DTPA and injected (100 µl) into an HPLC system equipped with a C18 column, Photodyode Array Detector (Waters 2996; for DHE) and fluorescence detectors. This allowed simultaneous detection of DHE and its derived oxidation products 2-hydroxyethidium (EOH, which detects preferentially superoxide) and ethidium (which detects less specific oxidants such as peroxides, as well as heme and peroxide activity), using DHE as an internal control during organic extraction of each sample [23]. Thus, DHE-derived products were expressed as ratios of EOH or ethidium generated per DHE consumed (initial minus remaining DHE concentration) [27].

### NADPH oxidase activity assay

Membrane fraction NADPH oxidase assays were performed as described previously [5], [26], [27]. Briefly, VSMC were disrupted by sonication in buffer containing Tris 50 mM, pH 7.4, EDTA 0.1 mM, EGTA 0.1 mM, and protease inhibitors (aprotinin 10 µg/mL, leupeptin 10 µg/mL and PMSF 1 mM) and centrifuged (18,000 g, 15 min). After supernatant centrifugation (100,000 g, 1 h), the obtained pellet (VSMC membrane fraction) was resuspended in the same buffer. To assess NADPH-triggered superoxide production, membrane homogenates (15 µg protein) were incubated with 10 µM DHE in phosphate buffer (50 mM, pH 7.4) with DTPA 0.1 mM in the presence of NADPH (50 µM) and DNA (1.25 µg/mL) for 30 min at 37°C in the dark. Fluorescence was followed (excitation/emission wavelengths for dihydroethidium: 490/590 nm) in a microplate spectrofluorometer (SpectraMax M5, Molecular Devices), as validated previously [27], [28].

### 20S proteasome proteolytic activity assay

20S proteasome activity (chymotrypsin-like) was measured with a Proteasome Assay Kit (EMD Biosciences) in VSMC homogenates stimulated or not with Angiotensin II (200 nM – used as a canonical Nox1 NADPH oxidase stimulus), Tn (5 µg/mL), MG132 (1 µM) or Tn+MG132 combinations for 16 h. This assay monitors the release of free AMC from the fluorogenic peptide Suc-Leu-Leu-Val-Tyr-AMC. The rate of AMC release was measured through the increase in fluorescence over time (excitation max.: 380 nm; emission max.: 460 nm). VSMC homogenates were obtained by cell lysis in RIPA buffer as described above.

### Quantitative PCR

Quantitative PCR was performed as described previously [28]. RNA was isolated with RNA SpinMini RNA isolation kit (GE Healthcare) and was converted to cDNA by incubation of 3 µg mRNA, 25 ng/L OligodT, 500 µM (each) dNTP, 5 µM dithiothreitol and SuperScript II (Invitrogen) at 42°C for 50 min. Quantitative PCR was performed with 150 ng of cDNA and Sybr Mastermix (Invitrogen) and was analyzed with Rotor-Gene 6000 Software (Corbett Research). Forward primers designed according to rabbit sequences were: Nox1 –CATCATGGAAGGAAGGAGA; Nox4 – CCACAGACTTGGCTTTGGAT; PDI – CGGCCCAGGAACTTCTTAAAGCCG; p22phox – GTACTTCATGGCGTAGGTGCCGAAGTAC.

### Western blot

VSMC homogenates were obtained by cell lysis in RIPA buffer, (Tris 20 mM, pH 8, NaCl 100 mM and glycerol 10%), with protease inhibitors (aprotinin 10 µg/mL, leupeptin 10 µg/mL, PMSF 1 mM) and Triton-100 10%. After 20 min on ice, samples were centrifuged (10,000 g, 10 min) and supernatants analyzed in SDS–PAGE as described [5], [27], [28]. Gel density was 12%, except for polyubiquitin gels (Fig.4), which was 8%. After protein transfer to nitrocellulose membrane, membranes were blocked with non-fat milk (5%, 2 h), blotted with primary antibodies overnight, and with secondary antibodies conjugated with horseradish peroxidase and luminescence was obtained by membrane incubation with chemiluminescence solution (luminol 2.5 mM, p-cumarinic acid 0.4 mM, H_2_O_2_ 5.4 mM, Tris 0.1 mM, pH 8.5). Densitometric analysis was performed with ImageQuant™ 2005 software (GE Healthcare).

Nuclear extracts used for the analysis of CHOP protein levels were obtained as reported [5]. Briefly, VSMC (∼1×10^6^ cells) grown in 10-mm dishes and exposed to experimental conditions were washed twice with cold PBS, scraped in 1 ml PBS-EDTA and transferred to 1.5 ml tubes. Cells were centrifuged at 2,500 rpm/5 min, pellets resuspended in 200 µl harvest buffer and incubated on ice for 5 min. Samples were centrifuged at 1000 rpm/10 min and nuclei pellet were washed twice with 500 µl buffer A (Hepes pH 7.0, 10 mM KCl, 0.1 mM EDTA, 0.1 mM EGTA) containing leupeptin (2 µg/ml), pepstatin (2 µg/ml), phenylmethylsulfonyl fluoride (44 µg/ml). Finally, pellets were resuspended in buffer C (10 mM HEPES, pH 7.9, 500 mM NaCl, 0.1 mM EDTA, 0.1 mM EGTA, 0.1% IGEPAL, containing leupeptin (2 µg/ml), pepstatin (2 µg/ml), PMSF (44 µg/ml). Samples were vigorously vortexed for 15 min at 4°C and after final centrifugation at 14000 rpm/10 min, supernatant containing 20 µg nuclear extracts was used for western blotting.

### XBP1 mRNA splicing

XBP1 mRNA splicing was detected according to published protocols [29]. Total RNA was extracted from 3.5×10^6^ VSMC incubated with or without Tn (5 µg/mL) and/or MG132 (1 µM) by using the Illustra mini RNA isolation kit (GE Healthcare). RT-PCR for samples was performed with Platinum Taq Polymerase from Invitrogen (Carlsbad, CA). PCR for XBP1 was performed with 3 µg cDNA and the primer sequences were AGAGAAAACTCATGGCCTTGTCATTG and GAAGAGTCAGCGCCGTCAGAA. PCR products were separated on a 3% agarose gel, which yielded a 238 bp product for unspliced and a 212 bp fragment for spliced XBP1 mRNA. PCR for GAPDH was performed under the same conditions with primer sequences TCACCATCTTCCAGGAGCG and CACAATGCCGAAGTGGTCGT, except that PCR products were separated on conventional agarose gels.

### Statistical analysis

Values are expressed as mean ± standard deviation. Statistical comparisons were performed through one-way analysis of variance (ANOVA), followed by Student–Newman Keuls test, at a 0.05 significant level (using The Primer of Biostatistics program, by Stanton A. Glantz, version 3.01, McGraw-Hill, 1992).
